# Expression patterns of mechanosensitive ion channel PIEZOs in irreversible pulpitis

**DOI:** 10.1186/s12903-024-04209-6

**Published:** 2024-04-16

**Authors:** Wenying Yang, Lu Lin, Shucheng Hu, Bin Jiang, Ruhan Yang, Weijun Yu, Jiaqi Tang, Dan Zhao, Yuting Gu, Min Jin, Jin Li, Eryi Lu

**Affiliations:** 1 Department of Stomatology, Renji Hospital, Shanghai Jiao Tong University School of Medicine, 160 Pujian Road, Shanghai, 200127 China; 2 Department of Ophthalmology, Renji Hospital, Shanghai Jiao Tong University School of Medicine, 160 Pujian Road, Shanghai, 200127 China

**Keywords:** Irreversible pulpitis, Mechanosensitive ion channels, PIEZO1, PIEZO2, Inflammation, Pain

## Abstract

**Background:**

Mechanosensitive ion channel PIEZOs have been widely reported to involve inflammation and pain. This study aimed to clarify expression patterns of PIEZOs and their potential relations to irreversible pulpitis.

**Materials and methods:**

Normal pulp tissues (*n* = 29) from patients with impacted third molars and inflamed pulp tissues (*n* = 23) from patients with irreversible pulpitis were collected. Pain levels were assessed using a numerical rating scale. PIEZO expressions were measured using real-time PCR and then confirmed using GEO datasets GSE77459, immunoblot, and immunohistochemistry staining. Correlations of PIEZO mRNA expression with inflammatory markers, pain markers, or clinical pain levels were evaluated using Spearman’s correlation analysis. Univariate analysis was conducted to analyze PIEZO expressions based on pain description and clinical examinations of cold test, percussion, palpation, and bite test.

**Results:**

Compared with normal pulp tissues, mRNA expression levels of PIEZO1 were significantly increased in inflamed pulp tissues, while PIEZO2 was significantly decreased, which was further confirmed in GSE77459 and on a protein and histological level. The positive correlation of the mRNA expression levels between PIEZO1 and inflammatory markers, as well as between PIEZO2 and pain markers, was verified. PIEZO2 expression was also positively correlated with pain levels. Besides, irreversible pulpitis patients who reported continuous pain and who detected a positive response to cold stimulus exhibited a higher expression level of PIEZO2 in the inflamed pulp tissues. By contrast, patients reporting pain duration of more than one week showed a higher expression level of PIEZO1.

**Conclusions:**

This study demonstrated the upregulation of PIEZO1 and the downregulation of PIEZO2 in irreversible pulpitis and revealed the potential relation of PIEZO1 and PIEZO2 to inflammation and pain. These findings suggested that PIEZOs might play critical roles in the progression of irreversible pulpitis and paved the way for further investigations aimed at novel therapies of irreversible pulpitis by targeting PIEZOs.

**Supplementary Information:**

The online version contains supplementary material available at 10.1186/s12903-024-04209-6.

## Background

Irreversible pulpitis, referring to the situation when the inflammation in pulp is not able to resolve, is a major dental disease affecting many adults. The inflammation in pulp results in pain, which is the most frequent chief complaint of irreversible pulpitis [1]. For patients, understandably, management of pain is given priority over all other considerations [2]. However, the unpredictable results of medications for inflammation and pain directly or indirectly limited the further development of treatment for irreversible pulpitis [3], implying an urgent need for a better understanding of irreversible pulpitis.

Pulpal inflammation could alter the dentinal sensitivity of nerve fibers and solicit varying degrees of pain. Inflammatory cytokines induced by invading pathogens could decrease the threshold of the nociceptors and cause pain through exciting or upregulating various ion channels mediating pain [4]. However, antibiotic drugs, non-steroidal drugs, and steroidal drugs aiming at arresting inflammation could erratically control pain [5–7]. Besides, local anaesthetics that block ion channels mediating pain may not achieve completely profound anaesthesia in patients with symptomatic pulpitis [8]. A better understanding of the pathological mechanism behind the disease is crucial for the improvement of treatment [9, 10]. Hence, exploring mechanisms underlying inflammatory pulpal pain may pave the way for novel therapies for irreversible pulpitis.

The mechanosensitive ion channel PIEZOs, PIEZO1 and PIEZO2, could convert mechanical signals to electrical or chemical signals by evoking cellular Ca^2+^ influx, and have been reported to participate in inflammatory processes and pain transmission [11]. For PIEZO1, more studies concentrated on its role in mechanical force-related inflammation. Reportedly, PIEZO1 could be upregulated by interleukin (IL) -1α, sense stiffness in macrophages and modulate their polarization, and mediate calcium-dependent injury and deformation microtrauma [12–14]. With regard to PIEZO2, accumulating evidence has shown its contribution to inflammatory pain, such as tactile allodynia and visceral hypersensitivity under inflammatory conditions [15–17]. Despite these important functions of PIEZOs in inflammatory diseases and pain, their potential role in irreversible pulpitis remains obscure.

Notably, emerging evidence has shown that PIEZOs might participated in the transduction of mechanical and sensory signals in pulp. PIEZO1 has been demonstrated to promote intercellular odontoblast-odontoblast and odontoblast-neuron communication and inhibit mechanically stimulated mineralization of odontoblast [18, 19]. PIEZO2 was found to be positive in dental primary afferent neurons with myelinated A-fibers and identified as a possible transducer for pulpal mechanical stimulation-activated inward currents [20]. Considering the contribution of PIEZOs in inflammatory diseases, it is implicated that PIEZOs may be involved in inflammatory pain in irreversible pulpitis.

Given that PIEZOs play a vital role in inflammation and pain, the current study assumed that PIEZO expressions were altered in irreversible pulpitis, and primarily compared expression patterns of PIEZO1 and PIEZO2 between irreversible pulpitis and normal pulp. To analyze their relations to inflammation and pain, the correlation of PIEZO expressions with the inflammatory mediators, IL-1β, tumor necrosis factor (TNF) -α, and IL-6 and pain markers, Neuropeptide Y (NPY) and tachykinin precursor 1 (TAC1) were assessed. This study also assessed the correlation between PIEZO expressions and clinical pain levels. Further, the expressional features of PIEZOs were analyzed based on pain description and clinical examinations of cold test, percussion, palpation, and bite test. Collectively, these results suggested the potential roles of PIEZO1 and PIEZO2 in inflammation and pain in irreversible pulpitis, which pave the way for further investigations aimed at clarifying the underlying mechanisms and achieving corresponding clinical outcomes for irreversible pulpitis.

## Materials and methods

### Sample collection

Twenty-nine normal pulp tissue samples were obtained from patients with impacted third molar teeth, and twenty-three inflamed pulp tissues were obtained from patients with irreversible pulpitis. The study was performed in accordance with the Declaration of Helsinki and received ethical approval from Ethics Committee of Renji Hospital Affiliated to Shanghai Jiao Tong University School of Medicine. Informed consent was obtained for experimentation with human subjects.

The clinical diagnosis of normal pulp and irreversible pulpitis is made according to the identification and definition of pulpal health and disease states approved by American Association of Endodontics Consensus Conference [1]. For patients with irreversible pulpitis, only those who developed irreversible pulpitis secondary to caries were included. For each patient, a standardized survey on the demographic information, history of dental pain (including pain categories, pain duration, and whether the pain was radiating), and medical/dental history were verbally administered. Tooth number was recorded, and examination procedures consisted of intraoral examinations to observe sinus tract, soft tissue, caries, and periodontal status, sensitivity cold tests, periapical tests to observe percussion, palpation and biting, and radiographs. Demographic information was sought by interviewing. Demographic and clinical information of the subjects was described in Table 1.

**Table 1:** Demographic and clinical information of the subjects

	Normal	Irreversible pulpitis
Age (mean ± S.E.M)	28.24 ± 1.60	31.00 ± 2.25
Male (%)	47.62	60.00
Female (%)	53.38	40.00
Pain levels (mean ± S.E.M)	0	4.20 ± 0.49
Intermittent pain (%)	0	46.67
Continuous pain (%)	0	53.33
Radiating pain (%)	0	26.67
Pain duration > 1 week (%)	0	40.00
Positive response to cold (%)	0	66.67
Percussion hypersensitivity (%)	0	46.67
Palpation hypersensitivity (%)	0	26.67
Bite test hypersensitivity (%)	0	80.00

*S.E.M* standard error of mean

Patients with the following situation were included for the normal group: (i) aged between 18 and 60; (ii) with impacted teeth and presenting for tooth extraction. Patients with the following situation were excluded: (i) any disease or medication history affecting pulpal status; (ii) oral or systemic diseases that would cause chronic or acute orofacial pain; (iii) mental health disorders, such as anxiety and depression, which cause pain; (iv) undergoing medical/dental treatment to manage pain; (v) smoking or drinking history; (vi) pregnant, lactating, or menopausal status.

Patients with the following situation were included for the diseased group: (i) aged between 18 and 60; (ii) diagnosed with irreversible pulpitis and presenting for endodontic treatment or tooth extraction with no evidence of periapical lesions and no previous pulp therapy. Patients with the following situation were excluded: (i) medication history affecting pulpal pain status; (ii) oral or systemic diseases that would cause chronic or acute orofacial pain; (iii) mental health disorders, such as anxiety and depression, which cause pain; (iv) undergoing medical/dental treatment to manage pain; (v) smoking or drinking history; (vi) pregnant, lactating, or menopausal status.

For RNA or protein isolation, normal pulp tissue samples were collected during extractions of impacted third molar teeth, and inflamed pulp tissue samples were obtained during pulpectomy. Subsequently, the pulp tissues were rinsed with normal saline, precooled, and stored using liquid nitrogen for further analysis. For histologic analysis, teeth from patients with extraction indications were extracted, rinsed with normal saline, and fixed in 10% neutral buffered formalin.

**Fig. 1:**
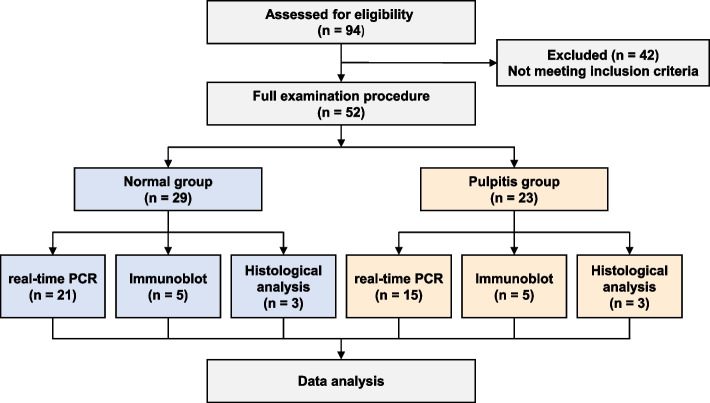
The STROBE flowchart

This laboratory study has been reported according to The Strengthening the Reporting of Observational Studies in Epidemiology (STROBE) statement: guidelines [21] (Fig. 1).

### Real-time Polymerase Chain Reaction (real-time PCR)

The mRNA expressions of *PIEZO1*, *PIEZO2*, *IL-1β*, *TNF-α*, *IL-6*, *NPY*, *CALCA*, *CALCB*, *TAC1*, and *ACTB* in the pulp tissues were measured using real-time PCR. Briefly, total RNA was isolated using TRIzol reagent (Invitrogen Life Technologies, Carlsbad, CA, USA) and qualified using NanoDrop Spectrophotometer (Thermo Fisher Scientific, USA). For each sample, 1-μg total RNA was added to a 20-μL reaction mixture using PrimeScript™ RT Master Mix kit (Takara Bio, Otsu, Shiga, Japan), and reverse-transcribed into cDNA using T100 Thermal Cycler (BIO-RAD, Hercules, CA, USA). Real-time PCR reaction mixture was prepared using FastStart Universal SYBR Green Master Mix reagent (Roche, Nutley, NJ, USA) and analyzed on a QuantStudio7 Flex Real-Time PCR System (Applied Biosystems, Foster City, CA, USA). Relative mRNA expression levels of *PIEZO1*, *PIEZO2*, *IL-1β*, *TNF-α*, *IL-6*, *NPY*, *CALCA*, *CALCB*, and *TAC1* were normalized to the mRNA level of human *ACTB* using the 2-DeltaDeltaCt method. Sequences of the primers are provided in Supplementary Table S1.

### Analysis of Gene Expression Omnibus (GEO) dataset GSE77459

GEO, a public genomics database of the National Center of Biotechnology Information, was thoroughly queried for datasets involving studies on inflamed pulp or pulpitis. The processed dataset of GSE77459 which measured the expression profile in human inflamed pulp and normal pulp tissues, was downloaded [22]. Data were analyzed in a log2 scale, and expression data on the probe level were transformed to gene level utilizing GPL17692.

### Immunoblot

Pulp tissues were frozen in radioimmunoprecipitation lysis buffer containing 1% phenylmethylsulfonyl fluoride, ground in liquid nitrogen, and lysed on ice for 30 min. Total protein concentration was detected using BCA Protein Assay Kit (Thermo Fisher Scientific, Rockford, IL, USA). Then sodium dodecyl sulfate loading buffer (Takara Bio, Otsu, Shiga, Japan) was added to protein samples, and the mixture was boiled at 100 ℃ for 5 min. Denatured protein samples were then loaded onto sodium dodecyl sulfate–polyacrylamide gels for separation and transferred onto polyvinylidene fluoride membranes. Membranes were blocked using bovine serum albumin blocking buffer for 1 h at room temperature, incubated in solutions containing primary antibodies against PIEZO1 (Novus Biologicals, MO, USA), PIEZO2 (Novus Biologicals), or GAPDH (Cell Signaling Technology, Danvers, MA, USA) overnight at 4 °C, washed with phosphate-buffered saline with Tween detergent, and then incubated in solution containing horseradish peroxidase-conjugated secondary antibody (Cell Signaling Technology) for 1 h at room temperature. After washing, membranes were exposed using chemiluminescence reagents (Millipore, Billerica, MA, USA) on ChemiDoc™ Imaging System (BIO-RAD, CA, USA). Immunoreactive bands were quantified using ImageJ software (X64; version 2.1.4).

### Histologic processing

Teeth were fixed adequately with 10% neutral buffered formalin. To reduce the time required for decalcification, the enamel parts of the fixed teeth were removed previously. Then the specimens were decalcified in 10% ethylene diamine tetraacetic acid, embedded in paraffin blocks, and cut into 2.5-μm sections. Subsequently, the sections were deparaffinized, hydrated and underwent Hematoxylin and eosin (H&E) staining or immunohistochemistry. For immunohistochemistry, briefly, the sections underwent antigen retrieval, blocking, antibody incubation, and visualization. Finally, the images were scanned and analyzed by a digital scanner (3DHISTECH, Pannoramic DESK, Budapest, Hungary).

### Pain intensity assessment

All the patients were asked to report the intensity of ongoing pain of the teeth immediately before the clinical treatment to collect the pulp tissues. The current pain levels were quantified using the 0–10 numerical rating scale (NRS), which has been reported previously [23]. In an NRS “0” denoted “no pain”, and “10” denoted “worst possible pain”.

### Statistical analysis

Data were analyzed using GraphPad Prism 9.0 and represented by mean ± standard error of mean (S.E.M). Significance of statistical differences between the normal pulp group and the inflamed pulp group was evaluated using the Mann–Whitney U test. The result was considered statistically significant when* P*-value was less than 0.05. The symbols of “*” indicated *P* < 0.05; “**” indicated *P* < 0.01; “***” *P* < 0.001 indicated *P* < 0.001. Correlations between mRNA expression levels were assessed using Spearman's correlation analysis. The absolute magnitude of Spearman's correlation coefficient *R*-value (R) was interpreted as follows: negligible (*R* = 0.00–0.10), weak (*R* = 0.10–0.39), moderate (*R* = 0.40–0.69), strong (*R* = 0.70–0.89), and very strong (*R* = 0.90–1.00) [24].

## Results

### Expression profile of PIEZO1 and PIEZO2 in normal and inflamed human pulp tissues

**Fig. 2:**
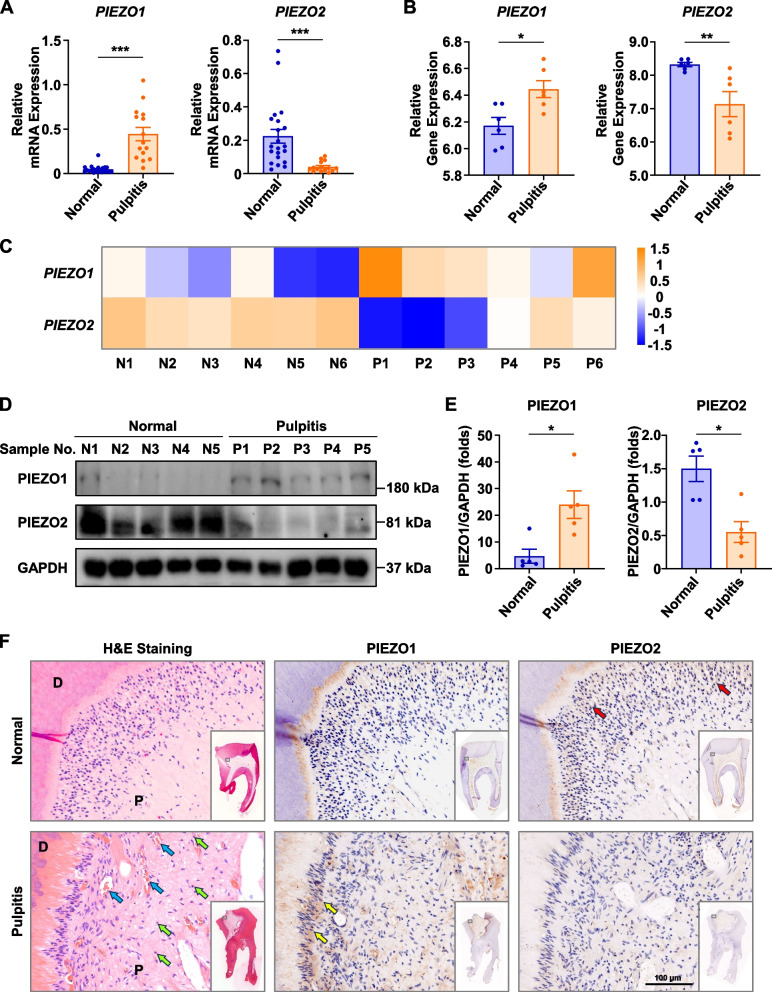
The expression patterns of Mechanosensitive ion channel PIEZOs in human irreversible pulpitis. **A** The mRNA expression levels of *PIEZO1* and *PIEZO2* in normal pulp tissues (*n* = 21) and inflamed pulp tissues (*n* = 15) were examined using real-time PCR. **B**, **C** The gene expression levels of *PIEZO1* and *PIEZO2* in normal pulp tissues (*n* = 6) and inflamed pulp tissues (*n* = 6) in the public database, GSE77459, were analyzed. **D** The protein expression levels of PIEZO1 and PIEZO2 in normal pulp tissues (*n* = 5) and inflamed pulp tissues (*n* = 5) were examined using immunoblot. **E** The immunoblot results of protein expression levels of PIEZO1 and PIEZO2 were quantified. **F** Representative images of H&E staining and immunohistochemistry of the pulp are presented. Inflammatory cells (green arrows) and more blood vessels (blue arrows) are shown in inflamed pulp using H&E staining. Highly expressed PIEZO1 (yellow arrows) and highly expressed PIEZO2 (red arrows) are shown in immunohistochemistry images. P, pulp; D, dentin. * represents *P* < 0.05; ** represents *P* < 0.01; *** represents *P* < 0.001

We first evaluated the mRNA expression levels of *PIEZO1* and *PIEZO2* in normal dental pulp tissues (*n* = 21) and inflamed pulp tissues (*n* = 15) using real-time PCR. The results showed that *PIEZO1* mRNA expression in the inflamed pulp tissues was significantly increased compared to normal pulp tissues, while *PIEZO2* mRNA expression was significantly decreased (Fig. 2A), which was in line with the analysis of the GEO dataset, GSE77459 (Fig. 2B, C). The upregulation of PIEZO1 and the downregulation of PIEZO2 in the inflamed pulp tissues were further validated using immunoblot (Fig. 2D, E). Besides, histological analysis was performed to examine the distribution of PIEZO expressions. H&E staining showed the infiltration of immune cells and more blood vessels in inflamed pulp tissues (Fig. 2F), which confirmed the inflammatory status in the pulp tissues. The results of immunohistochemistry staining indicated that the upregulation of PIEZO1 and the downregulation of PIEZO2 were mainly in the odontoblast layer (Fig. 2F). Overall, these data revealed that PIEZO1 expression increased while PIEZO2 expression decreased in irreversible pulpitis.

### PIEZO1 expression was positively correlated with IL-1β, TNF-α, and IL-6

**Fig. 3:**
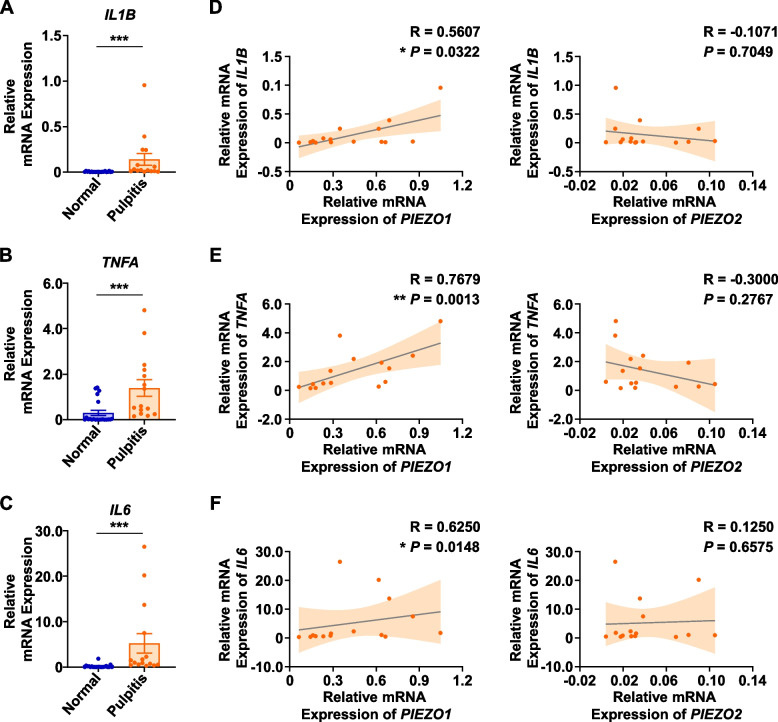
The mRNA expression levels of inflammatory markers *IL1B*, *TNFA*, and *IL6* and the correlations between PIEZOs and these inflammatory markers. **A**-**C** The mRNA expression levels of *IL1B*, *TNFA*, and *IL6* in normal pulp tissues (*n* = 21) and inflamed pulp tissues (*n* = 15) were presented, respectively. **D** The correlation of the mRNA expression levels between *PIEZO1* and *IL1B*, as well as *PIEZO2* and *IL1B*, was assessed using Spearman’s correlation analysis. **E** The Correlation of the mRNA expression levels between *PIEZO1* and *TNFA*, as well as *PIEZO2* and *TNFA*, was assessed using Spearman’s correlation analysis. **F** The Correlation of the mRNA expression levels between *PIEZO1* and *IL6*, as well as *PIEZO2* and *IL6*, was assessed using Spearman’s correlation analysis. * represents *P* < 0.05; ** represents *P* < 0.01; *** represents *P* < 0.001

IL-1β, TNF-α, and IL-6 are inflammatory mediators that have been widely reported to involve the inflammatory process and potentiate pain in pulpitis [25]. Expectedly, our results showed the significant upregulation of *IL1B*, *TNFA* and *IL6* in inflamed pulp tissues (Fig. 3A-C). Since we have demonstrated the alteration of PIEZO expressions in irreversible pulpitis, we then utilized Spearman’s correlation analysis and found that *PIEZO1* mRNA expression was significantly and positively correlated with *IL1B* (*R* = 0.5607, *P* = 0.0322, moderate), *TNFA* (*R* = 0.7679, *P* = 0.0013, strong), and *IL6* (*R* = 0.6250, *P* = 0.0148, moderate), but *PIEZO2* was not significantly correlated with *IL1B*, *TNFA*, or *IL6* (Fig. 3D-F). Therefore, our data revealed that PIEZO1 might be associated with inflammatory markers, implying that PIEZO1 presumably involved inflammatory processes in irreversible pulpitis.

### PIEZO2 expression was positively correlated with NPY and substance P

**Fig. 4:**
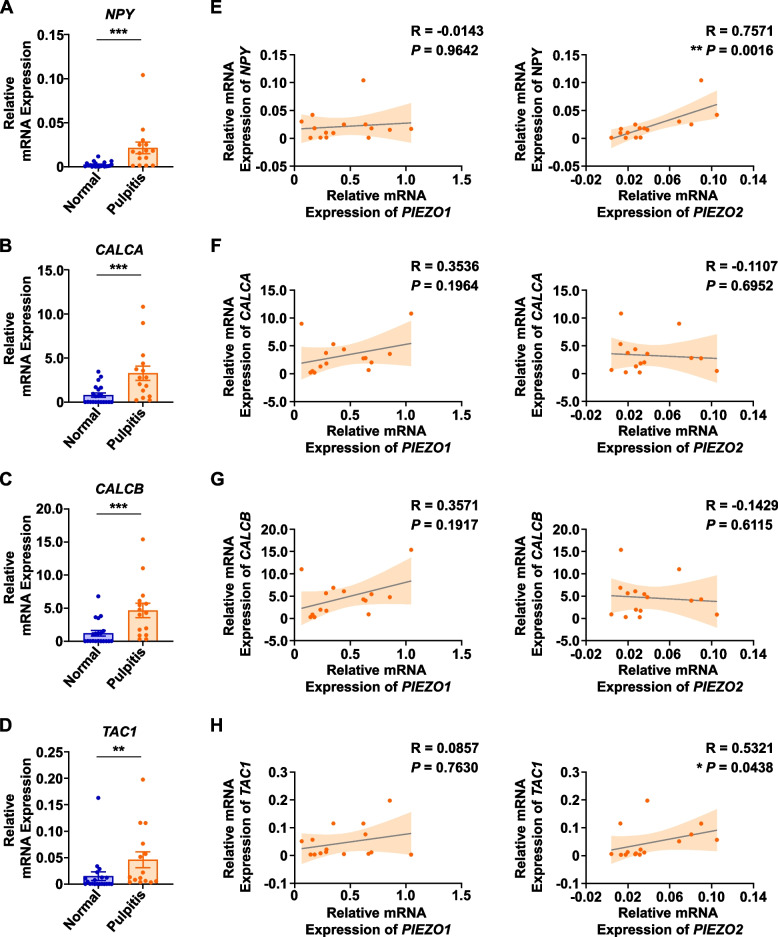
The mRNA expression levels of pain markers *NPY*, *CALCA*, *CALCB*, and *TAC1* and the correlations between PIEZOs and these pain markers. **A**-**D** The mRNA expression levels of *NPY*, *CALCA*, *CALCB*, and *TAC1* in normal pulp tissues (*n* = 21) and inflamed pulp tissues (*n* = 15) were presented, respectively. **E** The correlation of the mRNA expression levels between *PIEZO1* and *NPY*, as well as *PIEZO2* and *NPY*, was assessed using Spearman’s correlation analysis. **F** The correlation of the mRNA expression levels between *PIEZO1* and *CALCA*, as well as *PIEZO2* and *CALCA*, was assessed using Spearman’s correlation analysis. **G** The correlation of the mRNA expression levels between *PIEZO1* and *CALCB*, as well as *PIEZO2* and *CALCB*, was assessed using Spearman’s correlation analysis. **H** The correlation of the mRNA expression levels between *PIEZO1* and *TAC1*, as well as *PIEZO2* and *TAC1*, was assessed using Spearman’s correlation analysis. * represents *P* < 0.05; ** represents *P* < 0.01; *** represents *P* < 0.001

*NPY*, *calcitonin related polypeptide alpha* (*CALCA*), *calcitonin related polypeptide beta* (*CALCB*), and *TAC1* are genes that encode neuropeptides NPY, calcitonin gene-related peptide, and substance P, which are critical mediators of pain [25]. We first confirmed the upregulation of *NPY*, *CALCA*, *CALCB*, and *TAC1* in inflamed pulp tissues (Fig. 4A-D). Further, by using Spearman’s correlation analysis, we demonstrated the significant and positive correlation of *PIEZO2* expression with *NPY* (*R* = 0.7571, *P* = 0.0016, strong) (Fig. 4E) and *TAC1* (*R* = 0.5321, *P* = 0.0438, moderate) (Fig. 4H). However, no significant correlation was observed between *PIEZO1* and *NPY*, *CALCA*, *CALCB*, or *TAC1* (Fig. 4E-H). Therefore, *PIEZO2* might be related to pain mediator NPY and substance P, indicating the potential involvement of PIEZO2 in pain progression in irreversible pulpitis.

### PIEZO2 expression was positively correlated with clinical pain levels

**Fig. 5:**
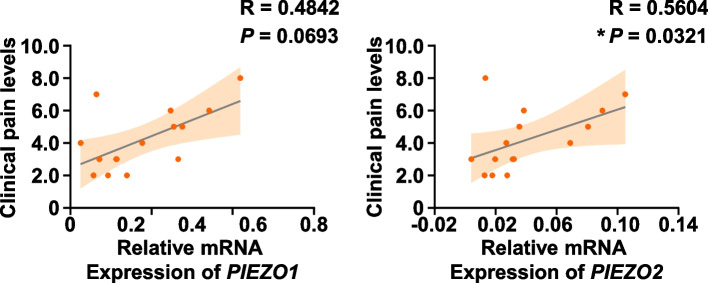
The correlation between *PIEZO1* mRNA expression level and pain levels, as well as *PIEZO2* and pain levels, was assessed using Spearman’s correlation analysis. * represents *P* < 0.05

In this study, the correlation between PIEZO expressions and pain levels was also performed using Spearman’s correlation analysis. As shown in Fig. 5, there was a significant and positive correlation between *PIEZO2* expression and pain levels of patients with irreversible pulpitis, but no significant correlation was found between *PIEZO1* and pain levels. In addition, univariate analysis was conducted to analyze PIEZO expressions based on the patient-reported pain description and responses to clinical examinations of cold test, percussion, palpation, and bite test (Table 2). Strikingly, *PIEZO2* expression was elevated in the pulp tissues from patients who reported continuous pain and who presented a positive cold response, while *PIEZO1* exhibited a significantly higher expression level in pulp tissues from patients who reported pain duration of more than one week. Collectively, these results indicated that PIEZOs might play a role in pain progression in irreversible pulpitis.

**Table 2:** Univariate analysis of PIEZO expressions

Variables		*PIEZO1* expression	*P*-value	*PIEZO2* expression	*P*-value
Intermittent/continuous pain	Intermittent	0.43 ± 0.10	> 0.999	0.02 ± 0.003	< 0.001***
	Continuous	0.46 ± 0.12		0.06 ± 0.01	
Pain duration	< 1 week	0.28 ± 0.06	0.007**	0.03 ± 0.01	0.181
	> 1 week	0.69 ± 0.10		0.05 ± 0.01	
Radiating pain	Radiating pain	0.42 ± 0.09	0.489	0.04 ± 0.01	0.490
	No radiating pain	0.51 ± 0.16		0.05 ± 0.02	
Cold test	Negative	0.32 ± 0.09	0.371	0.02 ± 0.004	0.013*
	Positive	0.50 ± 0.10		0.05 ± 0.01	
Percussion pain	Negative	0.35 ± 0.08	0.336	0.03 ± 0.003	0.190
	Positive	0.55 ± 0.13		0.06 ± 0.01	
Palpation pain	Negative	0.38 ± 0.07	0.280	0.03 ± 0.01	0.226
	Positive	0.63 ± 0.18		0.06 ± 0.02	
Bite pain	Negative	0.32 ± 0.06	0.734	0.03 ± 0.002	0.840
	Positive	0.47 ± 0.09		0.04 ± 0.01	

A *P*-value of < 0.05 is considered as significant. PIEZO1 expression and PIEZO2 expression are represented as mean ± standard error of mean

^*^represents *P* < 0.05

^**^represents *P* < 0.01

^***^represents *P* < 0.001

## Discussion

In this study, the upregulation of PIEZO1 and the downregulation of PIEZO2 were first confirmed in inflamed pulp tissues from patients with irreversible pulpitis. Consistently, chondrocytes in osteoarthritis cartilage displayed an increase in PIEZO1 expression upon IL-1α stimulation [12]. Besides, in cells exerted by mechanical stimulation, such as fluid sheer stress-treated osteocytes [26] and cyclic stretch-treated pulmonary microvascular endothelial cells [27], the increased expression of PIEZO1 was also observed. Hence, it is possible that the upregulation of PIEZO1 was a result of mechanical or inflammatory stimuli. Conversely, the reduced expression of PIEZO2 was demonstrated in irreversible pulpitis. Previous study has reported similar results that PIEZO2 was downregulated in pulmonary microvascular endothelial cells in response to abnormal shear stress, hypoxia, and TGF-β stimulation [28]. Considering that PIEZO2 has been widely reported to contribute to inflammation-induced sensitized mechanical pain [16], we speculated that the downregulation of PIEZO2 in inflamed pulp was a protective compensatory mechanism of the body to reduce harmful responses and relief pain, but it requires further validation.

We further demonstrated the positive correlation of the expression levels between PIEZO1 and inflammatory mediators, IL-1β, TNF-α, and IL-6. Reportedly, PIEZO1 could facilitate inflammation by transducing mechanical signals into inflammatory signals. For example, cyclical hydrostatic pressure could activate PIEZO1 in macrophages, thereby activating bacterial toxins-primed NOD-like receptor protein 3 inflammasomes and aggravating inflammation [29]. Besides, mechanical forces generated by cell adhesion molecule aggregation and blood flow could also activate PIEZO1 and induce downstream signalling events in endothelial cells, resulting in the opening of the endothelial barrier and leukocyte extravasation [30]. In infectious pulp tissues, cells could also experience mechanical force changes exerted by hydrostatic pressure changes or extravasation of leukocytes [23]. Therefore, PIEZO1 might also mediate the conversion of mechanical signals to inflammatory signals in irreversible pulpitis, but it requires further confirmation.

Herein, we also found that the expression level of PIEZO2 was positively correlated with pain markers, NPY and TAC1. PIEZO2 was demonstrated to be localized to low-threshold mechanoreceptors (LTMs) in sensory dorsal root ganglion neurons [31], which have been proven to produce substance P [32]. Besides, PIEZO2 was also localized to dental primary afferent neurons in trigeminal ganglions [20], which exhibited higher expression of LTM marker NPY [4]. Hence, future studies are required to study whether PIEZO2 acts on the function of LTMs to sense weak stimuli in inflamed pulp and whether it participates in the modulation of nociceptive neuropeptides.

Expectedly, the expression level of PIEZO2 was positively correlated with clinical pain levels, and PIEZO2 showed a higher expression level in patients with continuous pain and with positive responses to cold stimulation. This might be attributable to the potential association of PIEZO2 with nociceptive transmitters. Besides, PIEZO2 has been identified as a potential transducer to contribute to PIEZO2-like inward currents in dental primary afferent neurons [20] and mechanically activated currents of different kinetics in corneal trigeminal neurons [33]. Thus, it is also possible that PIEZO2 excitation could directly lead to subsequent inward currents to cause pulpal pain in pulpitis. By contrast, PIEZO1 exhibited elevated expression in irreversible pulpitis patients with pain duration of more than one week. As pain may come from inflammation, longer pain duration might reflect a longer inflammatory state of the pulp tissues. Combined with the correlation analysis that PIEZO1 expression was positively correlated with inflammatory mediators, IL-1β, TNF-α, and IL-6, it is speculated that PIEZO1 might play a vital role in the development of pulpal inflammation.

We also noticed that although PIEZO2 was found associated with pain in irreversible pulpitis, pain does not normally occur in normal pulp with higher expression of PIEZO2. This might be attributable to the functional activation of PIEZO2 under pathological conditions, which has indeed been observed previously. In dehydration, PIEZO2 was downregulated in kidney while PIEZO2 was significantly activated in juxtaglomerular renin-producing cells [34]. Besides, PIEZO2 has been demonstrated to be excited in nociceptive neurons under inflammatory conditions, although it was also highly expressed under normal conditions [35]. Evidence has shown that PIEZO2 mechanosensitivity could be potentiated by the cAMP pathway in inflammation [36]. Intriguingly, the quiescence of PIEZO2 was also observed in dental primary afferent upon mechanical stimulation under non-inflammatory conditions, by which approximately 43% of the PIEZO2-positive did not exhibit PIEZO2-like currents [20]. Hence, PIEZO2 might be functionally activated and mediate pain in irreversible pulpitis, but it requires further confirmation.

As the potential role of PIEZOs and their associations with inflammation and pain were indicated in this study, PIEZOs might serve as potential targets for irreversible pulpitis. Notably, the therapeutical effect of the non-specific inhibitor of PIEZO1, Gsmtx4, has been reported to ameliorate osteoarthritis in the animal model [37]. Gsmtx4 is also an inhibitor for PIEZO2 and could reduce adenosine 5'-triphosphate-induced Ca^2+^ influx in neuron tissues in-vitro, which indicated its therapeutic potential in mechanical allodynia [38]. Hence, the pharmacological inhibitors targeting PIEZOs may hold promise for novel therapies for irreversible pulpitis. However, small molecule inhibitors that selectively inhibit PIEZO1 or PIEZO2 are still in their infancy, and more in-depth studies and a clear theoretical basis to evaluate their safety are needed before the clinical trials.

## Conclusions

This study depicted the expression profile of mechanosensitive ion channel PIEZOs in pulp tissues from patients with irreversible pulpitis. The results identified the upregulation of PIEZO1 and the downregulation of PIEZO2. Furthermore, we verified the positive correlations of the mRNA expression levels between PIEZO1 and inflammatory mediators, IL-1β, TNF-α, and IL-6, as well as between PIEZO2 and pain markers, NPY and TAC1, in inflamed pulp tissues. We also demonstrated that PIEZO2 expression was positively correlated with pain levels. Besides, a higher expression level of PIEZO2 was found in the inflamed pulp tissues from the patients who described continuous pain and who responded to cold stimulus, while a higher expression level of PIEZO1 was found in patients with pain duration of more than one week. In summary, our findings provided new insights into the understanding of irreversible pulpitis pathogenesis, and future studies on PIEZOs and irreversible pulpitis might be helpful in leading to novel therapeutic approaches to relieve inflammation and pain in irreversible pulpitis.

### Supplementary Information

**Supplementary Material 1. **

**Supplementary Material 2. **

## Data Availability

The data and materials that support the findings of this study are available from the corresponding author upon reasonable request.

## Abbreviations

IL: Interleukin
TNF: Tumor necrosis factor
NPY: Neuropeptide Y
TAC1: Tachykinin precursor 1
STROBE: The Strengthening the Reporting of Observational Studies in Epidemiology
PCR: Polymerase Chain Reaction
GEO: Gene Expression Omnibus
H&E: Hematoxylin and eosin
S.E.M: Standard error of mean
CALCA: Calcitonin related polypeptide alpha
CALCB: Calcitonin related polypeptide beta
TAC1: Tachykinin precursor 1
LTM: Low-threshold mechanoreceptor
