# Supplementary material for: Expression patterns of mechanosensitive ion channel PIEZOs in irreversible pulpitis
Source: BMC Oral Health. 2024 Apr 16;24:465. doi: 10.1186/s12903-024-04209-6 (PMC11022356; doi:10.1186/s12903-024-04209-6)
Supplement: Supplementary file 1 — Supplementary Material 1. [file 12903_2024_4209_MOESM1_ESM.docx]

**Supplementary Table 1.** Primer sequences for Real-Time PCR.

| Gene | Primer | Sequence (5*'*→3*'*) |
| --- | --- | --- |
| *ACTB* | FW | CATGTACGTTGCTATCCAGGC |
|  | RV | CTCCTTAATGTCACGCACGAT |
| *PIEZO1* | FW | ATGTTGCTCTACACCCTGACC |
|  | RV | CCAGCACACACATAGATCCAGT |
| *PIEZO2* | FW | ACGACGATGCAAGGACATACG |
|  | RV | GCTCACCAACGTGATGTGG |
| *IL1B* | FW | AGCTACGAATCTCCGACCAC |
|  | RV | CGTTATCCCATGTGTCGAAGAA |
| *TNFA* | FW | TCTGGGCAGGTCTACTTTGG |
|  | RV | GGTTGAGGGTGTCTGAAGGA |
| *IL6* | FW | GCCACTCACCTCTTCAGAACG |
|  | RV | CAGTGCCTCTTTGCTGCTTTC |
| *NPY* | FW | CGCTGCGACACTACATCAAC |
|  | RV | CTCTGGGCTGGATCGTTTTCC |
| *CALCA* | FW | AAGCGGTGCGGTAATCTGAG |
|  | RV | GGGGAACGTGTGAAACTTGTTG |
| *CALCB* | FW | CACCTGTGTGACTCATCGGC |
|  | RV | GGGCACGAAGTTGCTCTTCA |
| *TAC1* | FW | GCAGAAGAAATAGGAGCCAATG |
|  | RV | CATAAAGAGCCTTTAACAGGGC |
