# Supplementary figures and images for: Expression patterns of mechanosensitive ion channel PIEZOs in irreversible pulpitis
Source: BMC Oral Health. 2024 Apr 16;24:465. doi: 10.1186/s12903-024-04209-6 (PMC11022356; doi:10.1186/s12903-024-04209-6)

Supplementary Figure 1

A

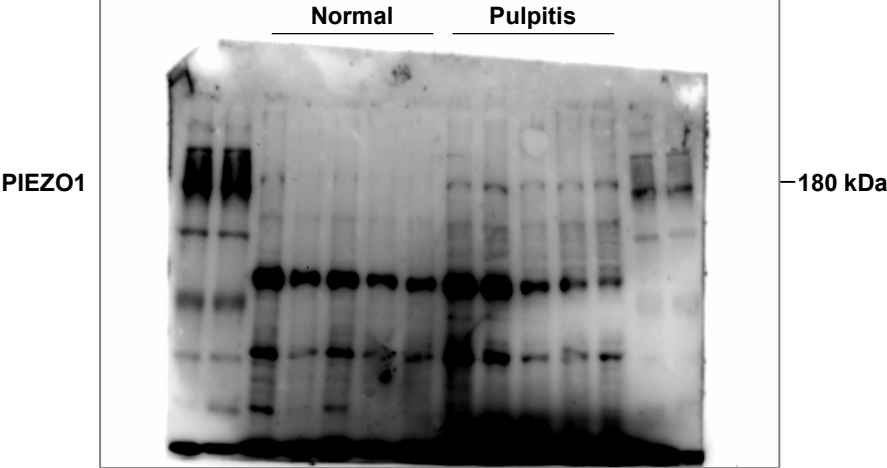

B

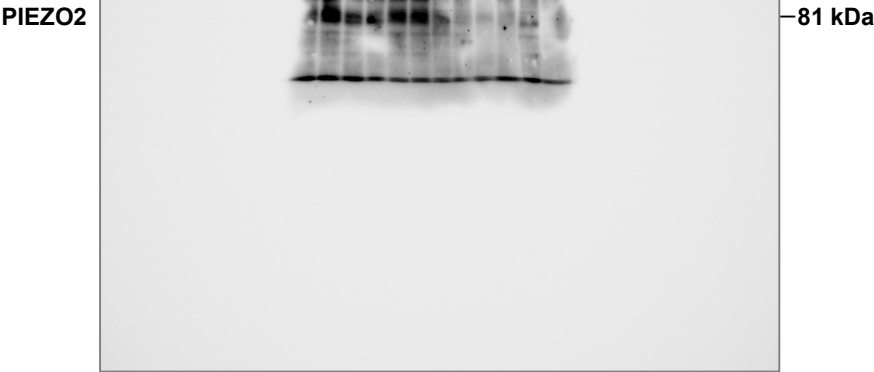

C

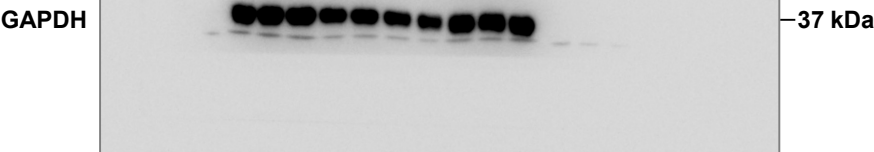

Supplement: Supplementary file 2 — Supplementary Material 2. [file 12903_2024_4209_MOESM2_ESM.pdf]
